# Urinary biomarkers for early diabetic nephropathy in type 2 diabetic patients

**DOI:** 10.1186/s40364-015-0042-3

**Published:** 2015-07-04

**Authors:** Temesgen Fiseha

**Affiliations:** Department of Clinical Laboratory Science, College of Medicine and Health Science, Wollo University, Dessie, Ethiopia

**Keywords:** Diabetic nephropathy, Microalbuminuria, Urinary biomarkers, Type 2 diabetes

## Abstract

Diabetic nephropathy (DN) is a serious complication of diabetes associated with increased risk of mortality, and cardiovascular and renal outcomes. Diagnostic markers to detect DN at early stage are important as early intervention can slow loss of kidney function and improve patient outcomes. Urinary biomarkers may be elevated in diabetic patients even before the appearance of microalbuminuria, and can be used as useful marker for detecting nephropathy in patients with normoalbuminuria (early DN). We reviewed some new and important urinary biomarkers, such as: Neutrophil gelatinase associated lipocalin (NGAL), N-acetyl-beta-glucosaminidase (NAG), Cystatin C, alpha 1-microglobulin, immunoglobulin G or M, type IV collagen, nephrin, angiotensinogen and liver-type fatty acid–binding protein (L-FABP) associated with early DN in type 2 diabetic patients. Our search identified a total of 42 studies that have been published to date. Urinary levels of these biomarkers were elevated in type 2 diabetic patients compared with non-diabetic controls, including in patients who had no signs indicating nephropathy (without microalbuminuria), and showed positive correlation with albuminuria. Despite the promise of these new urinary biomarkers, further large, multicenter prospective studies are still needed to confirm their clinical utility as a screening tool for early type 2 DN in every day practice.

## Introduction

Diabetic nephropathy is a severe complication occurring in diabetic patients and it is associated with an increased risk of all-cause mortality, cardiovascular disease and progression to end stage renal disease (ESRD), requiring costly renal replacement therapy in the form of dialysis or transplantation [1, 2]. Diagnostic marker to detect DN at early stage is important as early intervention can slow the loss of kidney function and reduce adverse outcomes. The appearance of small amount of protein albumin in urine, called microalbuminuria has been accepted as the earliest marker for development of DN. However, it has been reported that a large proportion of renal impairment occurs even before appearance of microalbuminuria [3]. Albuminuria has several confounding issues associated with it such as exercise, urinary tract infection, acute illness and cardiac failure. Furthermore, it has been reported to occur in the urine of non-diabetic subjects, indicating the non-specificity of albuminuria for accurate prediction of diabetic kidney disorder [4].

Given these shortcomings, additional urinary biomarkers that predict DN at a very early stage, even before the appearance of microalbuminuria are needed for optimal clinical management of diabetic patients. Several glomerular and tubular biomarkers predicting onset or progression of nephropathy in patients with diabetes have been identified and are becoming increasingly important in clinical diagnostics. Recent studies have demonstrated that urinary biomarkers were significantly elevated in normoalbuminuric type 2 diabetic patients compared with non-diabetic control subjects and could be used as markers for earlier, specific and accurate prediction of DN [4–6].

The interest for the use of biomarkers for early DN derives from the observation that patients with type 2 diabetes pass through a period of pre-diabetes and may experience renal impairment at the time of diagnosis. Although microalbuminuria has been considered as the earliest marker of DN in clinical practice, 29.1–61.6 % of individuals with type 2 diabetes could have renal impairment even before the onset of microalbuminuria, the gold standard for early diagnosis [7, 8]. A according to one study, type 2 diabetics with normoalbuminuric renal insufficiency were less likely to be identified as having any impaired kidney function as well as to have had their choice of drug type or drug dose adjusted compared to those with albuminuric renal insufficiency [9]. Thus, it is necessary to implement different strategies for detecting early DN in patients with type 2 diabetes aiming to delay its progression and improve outcomes. Increased levels of urinary biomarkers can be detected in type 2 diabetic patients before the onset of significant albuminuria and may be used as an early marker of renal injury in DN, this would play a significant role for the effective management and treatment approaches in diabetic care. The aim of this review is to summarize some new and important urinary biomarkers associated with early onset of DN and its progression in type 2 diabetic patients.

## Methods

This review is intended to explore aspects associated with the use of urinary biomarkers in early detection of renal damage, such as injury or dysfunction associated with DN in type 2 diabetic patients and it is not the intention of the authors to comment on the molecular function of the biomarkers. We performed a review of the literature with PubMed, US National Library of Medicine, Google Scholar, and Cochrane Library databases to determine if urinary biomarkers could be used to detect early DN. We used the following keywords: Urinary, Neutrophil gelatinase associated lipocalin, N-acetyl-β-D-glucosaminidase, Cystatin C, Alpha 1-microglobulin, Immunoglobulin G or M, Type IV collagen, Nephrin, Angiotensinogen and Liver-type fatty acid–binding protein (L-FABP), Normoalbuminuria, Diabetic nephropathy, and Type 2 diabetes. The keywords were searched alone or in combination with other keywords. We reviewed articles published between Jan 2002 and May 2015. Our search identified a total of 42 studies (only in human) that have been published to date.

### Neutrophil gelatinase associated lipocalin (NGAL)

Neutrophil gelatinase associated lipocalin (NGAL), an ubiquitous protein of approximately 25 kDa molecular mass, is produced and secreted into the urine in response to ischemic kidney damage and is therefore a promising early and sensitive biomarker of DN. The appearance of NGAL in the urine of patients may indicate early glomerular injury, and this has been demonstrated at earlier stage than the appearance of microalbuminuria, the traditional marker for early DN [10]. A more recent study reported that urine NGAL was raised early in diabetic and pre-diabetic nephropathy. Urine levels of NGAL were significantly higher in microalbuminuria group compared to normoalbuminuria and were positively correlated with urine ACR in both diabetes and pre-diabetes, suggesting that NGAL may play major role in development of nephropathy in pre-diabetes [11]. Another study found that urinary NGAL was significantly higher in type 2 diabetic patients than in controls and in micro- and macroalbuminuric than in normoalbuminuric patients, and was positively correlated to urinary albumin excretion [12]. Another study including type 2 diabetics with normo-, micro-, and macroalbuminuric DN found an increased urinary NGAL values with respect to controls. Increased levels of urinary NGAL were already found in diabetic patients without early signs of glomerular damage (normoalbuminuric), demonstrating usefulness of urinary NGAL as a marker of early DN. In the study, urinary NGAL was positively correlated with albuminuria and proteinuria, indicating that quantification of NGAL in urine will be a useful biomarker for reflecting the severity of renal damage caused by diabetic disease [13]. Another study showed that urinary NGAL was markedly increased in the type 2 diabetic patients compared with the controls, and was significantly increased from the normoalbuminuria to the last macroalbuminuria group. Urinary NGAL showed stronger positive correlations with urinary ACR and negative correlation with eGFR. The study suggested that tubular damage is common in short-term type 2 diabetic patients and urinary NGAL may be a promising early marker for monitoring renal impairment in this patients [14]. A follow-up study found an increasing tendency of urine NGAL in type 2 diabetics, from normo-albuminuria group to macro-albuminuria group, at both baseline and follow-up levels. Urine NGAL was found to be correlated positively with cystatin C, urea nitrogen and serum creatinine (SCr), and inversely with GFR, indicating that urinary NGAL could be used to predict the progression of DN in type-2 diabetic patients [15].

### N-acetyl-beta-glucosaminidase (NAG)

The urinary enzyme N-acetyl-beta-glucosaminidase (NAG) is found in the lyzozomes of the proximal tubule epithelial cells, and a high NAG activity in urine may indicate an early sign of renal disorder. In one study, urinary NAG activities were found to be elevated in normoalbuminuric type 2 diabetic patients as compared to controls, and correlated inversely with measured and estimated creatinine clearance. The receiver operating characteristic (ROC; diagnostic accuracy) plot of this study showed that urinary NAG had higher sensitivity than SCr and met the criteria for detecting glomerular and tubular dysfunction as screening tests for early diagnosis of DN, demonstrating usefulness of urinary NAG as a biomarker for early DN in diabetes [16]. Another study from type 2 diabetic patients found that urinary NAG was significantly higher in all patient groups than in controls and in microalbuminuric than in normoalbuminuric patients [12]. In another study, urinary NAG was higher in 34 % patients at the normoalbuminuric stage, 63.7 % at the microalbuminuric stage and 49.5 % overall diabetic cases, which indicated that urinary NAG excretion might be a useful biomarker of early renal injury in diabetic patients, even at the normoalbuminuric stage [17]. A more recent study found that urinary NAG was significantly higher in type 2 diabetic patients with normo-, micro- and macroalbuminuria than in non diabetic controls, and its value increased in parallel with the severity of renal involvement. Significant positive correlation was observed between urinary NAG and ACR, SCr and HbA1c, suggesting that urinary NAG could be used as a useful biomarker reflecting the degree of renal impairment in DN [18]. Another study showed that urinary NAG levels were significantly increased in microalbuminuria group compared to normoalbuminuria group and correlated positively with ACR, indicating the possible clinical application of urinary NAG as a complementary marker for early detection of DN in type 2 diabetes [19].

### Cystatin C

Cystatin C, a cysteine protease inhibitor constantly produced by all nucleated cells, has been suggested as a marker of glomerular and tubular dysfunction for early diagnosis of DN [16]. Urine levels of cystatin C were significantly higher in microalbuminuria group compared to normoalbuminuria and were positively correlated with urine ACR in both diabetes and pre-diabetes. Urine cystatin C was raised early in diabetic and pre-diabetic nephropathy, suggesting that cystatin C may play major role in development of nephropathy in pre-diabetes [11]. One study showed that cystatin C levels of urine might be a marker of early renal damage among patients with type 2 diabetes mellitus [20]. Urinary levels of cystatin C were significantly increased in patients with microalbuminuria without any other urinary abnormality and with normal SCr as compared to those without microalbuminuria or any other urinary abnormality and showed a positive correlation with urinary ACR, which indicated that urinary cystatin C might be a novel biomarker of early DN [21]. Another study from type 2 diabetic patients found that increased urinary cystatin C was associated with decline in GFR, particularly at the early stages of DN in patients with an eGFR of ≥ 60 mL/min/1.73 m^2^, suggesting that higher urinary cystatin C excretion was a better predictor of early nephropathy [22]. In another study, urinary cystatin C levels were identified as an independent factor associated with eGFR < 60 ml/min/1.73 m^2^ in patients with normal albuminuria excretion. The cystatin C levels of urine increased with increasing degree of albuminuria, reaching higher levels in macroalbuminuric patients, demonstrating usefulness of urinary cystatin C as a biomarker for detecting onset of nephropathy in type 2 diabetic patients with normoalbuminuria (early nephropathy) [23]. All these data suggest that cystatin C is a promising biomarker for early DN in type 2 diabetics; however, further studies are required to document the clinical utility of urinary cystatin C for early DN.

### Alpha 1-microglobulin

Alpha 1-microglobulin (α1-microglobulin) is a 27-kDa glycoprotein which is filtered freely by the glomeruli and reabsorbed by the proximal tubule. Increased urinary excretion of α1-microglobulin has been suggested as an early biomarker for screening DN. In one study, raised urinary α1-microglobulin was found in 33.6 % of patients with normoalbuminuria, 53.6 % microalbuminuria and 64.5 % macroalbuminuria, suggesting that urinary α1-microglobulin might be useful for the early detection of DN in diabetics. Urinary α1-microglobulin was related to duration, severity and control of diabetes, and was directly related to albuminuria, indicating that it is a good biomarker of the severity of renal impairment in type 2 diabetic patients [24]. In another study, 45.2 % had elevated urinary α1-microglobulin excretion, 23.1 % microalbuminuria, 9.6 % macroalbuminuria and 27.2 % had a eGFR < 60 mL/min/1.73 m^2^, which indicated that urinary α1-microglobulin might be a useful biomarker for detecting early DN in type 2 diabetes, even before albuminuria and eGFR [8]. Another study found that urinary α1-microglobulin was significantly higher in patients as compared to controls and in microalbuminuric than normoalbuminuric groups. Urinary α1-microglobulin was correlated with urinary ACR even at high-to-normal levels, demonstrating usefulness of urinary α1-microglobulin as a biomarker for early DN in type 2 diabetic patients [25]. In another study, 27.9 % and 23.5 % of normoalbuminuric patients with type 2 diabetes mellitus were found to have increased urinary α1 - and β2 – microglobulin levels, respectively [26]. Thus, urinary α1-microglobulin enables noninvasive assessment of DN development at an early stage, while more studies are needed to confirm its utility in type 2 DN.

### Immunoglobulin M/G

Immunoglobulin G or M (IgG or IgM) are large proteins (mol radius 5.5 and 12 nm, respectively) synthesized and secreted by plasma cells, and their appearance in urine indicates that a large, nonselective pore exists in the glomerular capillary wall. One study indicated that type 2 DN patients had a significantly higher degree of albuminuria and many-fold higher urinary IgG and urinary IgM concentrations than the healthy control subjectes [27]. Another study found that urinary levels of IgM and IgG2 were elevated in 47 and 50 patients while only 21 patients had albuminuria, and 12 patients had a ratio of IgG2/IgG4 < 1. Elevated urinary levels of IgM and IgG2 indicated decreased glomerular size selectivity; while a ratio of IgG2/IgG4 < 1 indicated decreased charge selectivity. The study suggested that elevated urinary levels of IgM and IgG2 might be more sensitive markers of renal disease than albuminuria in patients with type 2 diabetes and antihypertensive therapy [28]. Another study showed that the urinary excretion rates of IgG, ceruloplasmin, transferrin, and orosomucoid, were significantly higher in normoalbuminuric type 2 diabetic patients than in non-diabetic control subjects [29]. Another study found that urinary IgG excretion was increased significantly in diabetic patients compared to healthy controls and was further increased significantly in chronic renal failure patients with respect to the clinical stage of nephropathy. The increase in urinary IgG excretion was radically increased in diabetic patients with nephropathy compared to other groups and was 75 % higher in DN patients as compared in the non-DN patients, suggesting the significant role of urinary IgG excretion as a biomarker for early type 2 DN [30]. A follow-up study reported that increased urinary-IgG excretion could predict development of microalbuminuria in normoalbuminuric type 2 diabetic patients. The rate of progression to microalbuminuria was significantly higher in patients with increased urinary-IgG (47.1 %) than in patients without increased urinary-IgG (9 %), which indicated urinary IgG levels could be a useful biomarker for development of DN in normoalbuminuric type 2 diabetic patients [31].

### Type IV collagen

Type IV collagen is the principal component of glomerular basement membrane and messangial matrix and its excretion in urine might serve as early indicator of renal injury associated with DN. One study found that urinary type IV collagen levels were significantly higher in the normoalbuminuric group with diabetes than in the control group. Urinary type IV collagen significantly increased in microalbuminuric patients compared to patients without microalbuminuria, and levels were positively correlated with the ACR, suggesting that urinary type IV collagen might be useful as a early marker of DN in type 2 diabetic patients [32]. Another study found that urinary type IV collagen levels were higher in patients with microalbuminuria than in those with normoalbuminuria and correlated with urinary albumin excretion rate, which indicated that urinary type IV collagen might be useful in predicting early DN in type diabetes [33]. Another study found that urinary type IV collagen excretion was significantly increased in type 2 diabetics, in both normoalbuminuric and microalbuminuric patients, compared with healthy controls and correlated with the amount of urinary albumin [34]. Another study from type 2 diabetic patients showed that urinary collagen IV levels were significantly higher in patients compared to healthy subjects and were significantly higher in micro- and macroalbuminuric groups than normoalbuminuric group. Urinary collagen IV levels correlate positively with ACR and inversely with eGFR, suggesting that urinary type IV collagen may be considered as noninvasive and early marker for DN before microalbuminuria stage and as an indicator of DN progression [35]. A follow-up study showed that 76.8 % of normoalbuminuric type 2 diabetic patients in the high urinary type IV collagen group developed microalbuminuria as opposed to 22.6 % patients in the normal urinary type IV collagen group. This indicated that baseline urinary type IV collagen excretion may be an independent predictive factor for the development of DN in normoalbuminuric type 2 diabetic patients [36].

### Nephrin

Podocytes are key structural elements of the glomerular filtration barrier, and their detachment may play important role in the development and progression of DN in type 2 diabetes [37]. A study suggested that diabetes subjects had higher urinary levels of podocyte-associated proteins than non-diabetic subjects, even the normoalbuminuric patients, and might be used as potential biomarkers for early renal injury in diabetes [38]. Nephrin is a 180 KD trans-membrane protein expressed in glomerular podocytes and its presence in the urine correlates with podocyte lesions in the course of DN. A systemic review, including 19 studies, indicated that urine nephrin analysis has the potential to become an important biomarker of early glomerular injury [39]. Another systemic review suggested that the presence of nephrin in urine can offer a higher sensitivity and specificity for earlier detection of DN and the levels of which can also ascertain the severity of the disease [40]. In the above study, nephrinuria was found in 53 %, 71 %, and 90 % of normo-, micro-, and macroalbuminuric diabetes subjects, respectively. Urinary excretion of nephrin was higher in diabetics than in non diabetics and correlated with increasing albuminuria [38]. Another study showed that nephrinuria was increased in patients with type 2 diabetes, even in the normoalbuminuria stage, and levels were correlated with urinary ACR and the eGFR, suggesting that nephrin measurement in urine offers the possibility for early detection of DN [25]. Another study found that nephrinuria was significantly associated with lower eGFR even among normoalbuminuric type 2 diabetic patients, which indicated that urinary nephrin excretion may be involved in the development of DN in the stage of normoalbuminuria [41]. Another study found that nephrinuria was detected in 100 % of patients with micro- and macroalbuminuria, as well as 54 % of patients with normoalbuminuria. They also found the highest correlation between urinary nephrin levels with albuminuria and negative correlations with eGFR, suggesting its potential utility as an early biomarker of DN in type 2 diabetic patients [42].

### Angiotensinogen

Angiotensinogen is synthesized primarily in cells of the proximal tubule and is secreted from the apical surface into the lumen, where it is converted first to angiotensin (Ang) I and then Ang II by tubular renin and angiotensin-converting enzyme (ACE), respectively. It has been suggested that urinary angiotensinogen levels can function as early biomarkers for determining the development and progression of DN. One study found that urinary angiotensinogen was higher in the micro- and macroalbuminuric patients compeered to controls and in normoalbuminuric patients than in controls, indicating that angiotensinogen could be used as a potential urinary biomarker for kidney injury in albuminuric and non-albuminuric conditions. Significant differences were found between the urine angiotensinogen values in each stage of DN. Urinary angiotensinogen correlated significantly with urinary ACR, suggesting that urinary angiotensinogen levels were associated with severity or exaggerated renal function in patients with type 2 diabetes [43]. Another study reported that urinary angiotensinogen is well correlated with urinary α1-microglobulin and reflected the tubular injuries which may be associated with the intrarenal renin–angiotensin system (RAS) activation in patients with type 2 diabetes [44]. A follow-up study reported that urinary angiotensinogen levels correlated with urinary ACR and urinary β2- microglobulin and inversely with eGFR. Follow-up analysis of this study indicated that patients with albuminuria and high levels of urinary angiotensinogen showed the progressive decline of renal function and the high incidence of renal-cardiovascular endpoints. From this, the study suggested that the higher level of urinary angiotensinogen in type 2 diabetic patients with nephropathy is a high risk factor for worsening renal and cardiovascular complications [45]. Another study found increased ACE2 enzyme activity in urine from both albuminuric and non-albuminuric type 2 diabetic patients as compared to healthy controls, suggesting urinary ACE2 may function as an early marker of DN. Urinary ACE2/Cr levels were much higher in hypertensive diabetic patients compared with their normotensive counterparts and treatment with RAS inhibitors markedly suppresses urinary ACE2 excretion. This indicated that, in addition to its role as a marker of DN, urinary ACE2 might potentially function as a marker for monitoring the therapeutic response of RAS inhibitors in diabetes [46]. Urinary angiotensinogen enables noninvasive assessment of DN risk in type 2 diabetics at an early stage, while more studies are needed to investigate the role of urinary angiotensinogen in type 2 DN.

### Liver-type fatty acid–binding protein (L-FABP)

Liver-type fatty acid–binding protein (L-FABP) is a 14 kDa protein that is expressed in proximal tubular cells of the kidney. Evidence suggests that urinary excretion of L-FABP might be a suitable early biomarker for predicting the onset of DN and its progression in diabetics. One more recent study suggested that elevated levels of urinary L-FABP are evident from the microalbuminuric stage indicating tubular damage in type 2 diabetic patients. Urinary L-FABP levels were elevated in patients with reduced eGFR and showed a positive correlation with protein to creatinine ratio [47]. Urinary L-FABP was significantly increased in type 2 diabetic patients compared with healthy controls and correlated with albumin excretion rate, creatinine clearance and haemoglobin levels, suggesting its ability as a novel biomarker of early DN and chronic intrarenal ischaemia [48]. Another study indicated that the level of urinary L-FABP accurately reflected the severity of DN and was significantly higher in the patients with type 2 diabetes who had normoalbuminuria than in normal control subjects. Urinary L-FABP higher than the upper limit of reference value was a risk factor for progression of DN, which indicated that urinary L-FABP could be a useful marker for the detection of early-stage DN and for the prediction of the progression of DN [49]. A long term observational study on type 2 diabetic patients without advanced nephropathy revealed that higher levels of urinary L-FABP were associated with deteriorating renal function and a higher incidence rate of CVD. This association was markedly observed even in patients with normoalbuminuria, suggesting that urinary L-FABP can be used as a biomarker for predicting future renal dysfunction and incidence of CVD in type 2 diabetic patients with an early stage of nephropathy, in addition to albuminuria [50]. Another study suggested that simultaneous measurement of urinary L-FABP and ACR should be useful to assess cardiovascular damage reflecting on the elevation of cardiac markers and electrocardiographic (ECG) abnormalities in type 2 diabetic patients with CKD stage G1 and G2 [51]. All these data suggest that urinary L-FABP is a promising early biomarker of DN and incidence of important health outcomes in type 2 diabetes, while further studies are needed to investigate the role of urinary L-FABP in type 2 DN.

## Conclusions

Microalbuminuria has been accepted as the earliest marker for diabetic nephropathy; however, a large proportion of renal impairment occurs in non-albuminuric state. It is important to implement different strategies for earlier detection of DN aiming to prevent the long-term devastating outcomes of renal loss in diabetics. Urinary biomarkers were significantly elevated in normoalbuminuric type 2 diabetic patients compared with non-diabetic control subjects and could be used as markers of DN at a very early stage even before the development of microalbuminuria, the current gold standard for early diagnosis. Despite the promise of these new biomarkers, further large, multicenter prospective studies are still needed to confirm their clinical utility as a screening tool for every day practice.

## Abbreviations

ACR: Albumin: creatinine ratio
DN: Diabetic nephropathy
eGFR: Estimated glomerular filtration rate
IgG: Immunoglobulin G
IgM: Immunoglobulin M
L-FABP: Liver-type fatty acid–binding protein
NAG: N-acetyl-beta-glucosaminidase
NGAL: Neutrophil gelatinase-associated lipocalin
